# A Case of Castration for Hypertrophied Prostate

**Published:** 1897-06

**Authors:** 


### A CASE OF CASTRATION FOR HYPERTROPHIED PROSTATE

Is related, by Chevalier, of Paris, where supra-pubic punctures, cystotomy and resection of both vasa had been previously performed.

Patient, 59 years old, had several acute attacks of retention of urine. During October, 1895, bladder was tapped repeatedly, catheterization being impossible, with negative results.

Cystotomy was then performed November 1, 1895, and five weeks later the urethra was still impervious; both vasa were resected (December 6, 1895).

March 12, 1896—Prostate very large, the urethra barely permeable and a vesical fistula was noted. Patient desired to be relieved of his hypogastric meatus.

April 29, 1896—Castration with simultaneous closure of supra-pubic fistula; catheter left in bladder until fistula was closed.

June 3, 1896—The patient left the hospital, emptying his bladder naturally, but every half hour. The prostate had, however, decreased one-third.

September 3, 1896—Patient voids a clear and odorless urine about every three hours.—*Revue de Chirurgie.*

